# Psychiatry training in Hungary, difficulties and advantages

**DOI:** 10.1192/j.eurpsy.2024.1657

**Published:** 2024-08-27

**Authors:** I. A. Van Der Wijk

**Affiliations:** Sántha kálmán Member Hospital, SzSzBM County Hospital, Nagykálló, Hungary

## Abstract

**Introduction:**

While in theory our training program is quite satisfactory, in practise it often falls short. The first two years give a more general knowledge, including spending time at internal, ICU and neurological wards as well as attending a month-long course about communication, palliative care and basic legal principals important in healthcare. The second three years provide the opportunity to engage in profession-related rotations, like psychotherapy, psychiatric rehabilitation and addictology.

**Objectives:**

The design in itself is clear, but the supervision for its enactment is insufficient. This leads to regional differences between the four faculties of our country, not everyone is able to partake in the supposedly mandatory rotations (mostly because of shortcomings in staff ) and the organization of our theoretical education varies greatly in each region to the point of non-existence in one area, since the COVID-19 pandemic started. The personal supervision of each psychiatry trainee also leaves much to be desired both on professional and – in psychiatry very important – mental levels. Competence and responsibility limits are often vague, and, especially in country hospitals, to much is expected of the resident (i.e. doing a nightshift alone, without direct supervision).

**Methods:**

It is a positive thing that in theory there are standards in place, the problem is that they are more viewed as guidelines, than demands to be met. Nevertheless, some of the faculties provide well-organized education (even subdivided per year of training) and/or take rotations outside of the ‘home ward’ seriously. The opportunity to gain a basic knowledge in psychotherapy is also beneficial and a good aspect of our training. Easily accessible or even obligatory participation in psychotherapy for ourselves during our training however, is lacking.

**Results:**

The decreasing number of psychiatry trainees sadly is a worldwide trend and Hungary is no exception. This poses more difficulties, i.e. making it harder to let a resident go on ‘outside’ rotations, especially from wards already struggling with staff shortages. Because of the latter, there is also little time to teach the trainees appropriately and pay them the attention they need.

**Conclusions:**

All in all, there is much potential in our training program and its standards, also leaving room for substantial improvement in realizing the practical aspects. The decline in numbers of psychiatry trainees is worrisome and calls for more general intervention on a European or even global level.

**Disclosure of Interest:**

None Declared

